# Bis(benzoato-κ^2^ *O*,*O*′)(2,2′-bipyridine-κ^2^ *N*,*N*′)lead(II) benzoic acid monosolvate

**DOI:** 10.1107/S1600536810046489

**Published:** 2010-11-17

**Authors:** Juan Yang, Jiantong Li

**Affiliations:** a Department of Physical Chemistry, Henan Polytechnic University, Jiaozuo 454003, People’s Republic of China

## Abstract

The reaction of lead acetate, benzoic acid and 2,2′-bipyridine (bipy) in aqueous solution yielded the title complex, [Pb(C_7_H_5_O_2_)_2_(C_10_H_8_N_2_)]·C_7_H_6_O_2_. The asymmetric unit contains two independent complex mol­ecules as well as two independent benzoic acid solvent mol­ecules, one of which is disordered over two positions with almost equal occupancies [0.504 (5) and 0.496 (5)]. The two complex mol­ecules have similar configurations with the hexa­coordinated environment of the Pb^II^ atom formed by four carboxyl­ate O atoms of two chelate benzoate ligands and two N atoms of the bipy ligand. The Pb—O bonds involving one of the benzoate ligands are almost coplanar with Pb—N bonds to the bipy ligand [dihedral angles of 12.67 (11) and 14.73 (11)°] ; if the second benzoate ligand is treated as one coordination site, the overall coordination may be represented as a distorted pseudo-square pyramid. Weak inter­molecular Pb⋯O inter­actions [3.046 (3) and 3.359 (3) Å] link each of the complex mol­ecules into two symmetry-independent centrosymmetric dimers. Hydrogen bonds involving the carboxyl H atoms of solvent benzoic acid mol­ecules and metal-coordinated carboxyl­ate O atoms link complex mol­ecules and benzoic acid solvent mol­ecules into insular aggregates.

## Related literature

For potential applications of Pb(II) complexes, see: Fan & Zhu (2006 ▶); Hamilton *et al.* (2004 ▶); Alvarado *et al.* (2005 ▶). For the use of aromatic carboxyl­ates and 2,2′-bipyridine-type ligands in the preparation of metal complexes, see: Wang *et al.* (2006 ▶); Masaoka *et al.* (2001 ▶).
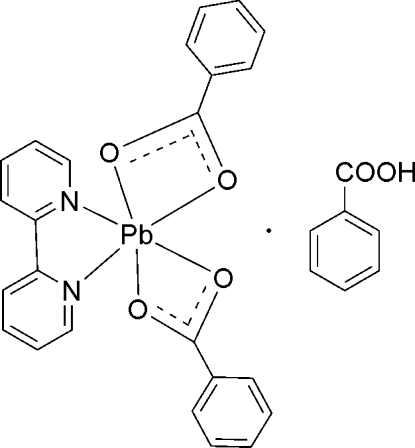


## Experimental

### 

#### Crystal data

[Pb(C_7_H_5_O_2_)_2_(C_10_H_8_N_2_)]·C_7_H_6_O_2_
*M* *_r_* = 727.71
Triclinic, 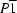

*a* = 9.6298 (2) Å
*b* = 10.4264 (2) Å
*c* = 28.7365 (5) Å
α = 84.843 (1)°
β = 88.128 (1)°
γ = 72.619 (1)°
*V* = 2742.32 (9) Å^3^
*Z* = 4
Mo *K*α radiation
μ = 6.2 mm^−1^
*T* = 296 K
0.24 × 0.19 × 0.15 mm

#### Data collection

Bruker APEXII CCD area-detector diffractometer
Absorption correction: multi-scan (*SADABS*; Bruker, 2007 ▶) *T*_min_ = 0.253, *T*_max_ = 0.395
49796 measured reflections
14302 independent reflections
10344 reflections with *I* > 2σ(*I*)
*R*_int_ = 0.039

#### Refinement

*R*[*F* ^2^ > 2σ(*F* ^2^)] = 0.035
*wR*(*F* ^2^) = 0.081
*S* = 1.01
14302 reflections
773 parameters
H-atom parameters constrained
Δρ_max_ = 1.17 e Å^−3^
Δρ_min_ = −1.13 e Å^−3^

### 

Data collection: *APEX2* (Bruker, 2007 ▶); cell refinement: *SAINT* (Bruker, 2007 ▶); data reduction: *SAINT*; program(s) used to solve structure: *SHELXS97* (Sheldrick, 2008 ▶); program(s) used to refine structure: *SHELXL97* (Sheldrick, 2008 ▶); molecular graphics: *SHELXTL* (Sheldrick, 2008 ▶); software used to prepare material for publication: *SHELXTL*.

## Supplementary Material

Crystal structure: contains datablocks global, I. DOI: 10.1107/S1600536810046489/ya2132sup1.cif

Structure factors: contains datablocks I. DOI: 10.1107/S1600536810046489/ya2132Isup2.hkl

Additional supplementary materials:  crystallographic information; 3D view; checkCIF report

## Figures and Tables

**Table 1:** Hydrogen-bond geometry (Å, °)

*D*—H⋯*A*	*D*—H	H⋯*A*	*D*⋯*A*	*D*—H⋯*A*
O10—H10⋯O5	0.85	1.83	2.670 (5)	171
O12*A*—H12*C*⋯O9^i^	0.85	1.62	2.459 (9)	169
O12*B*—H12*B*⋯O4^ii^	0.85	1.81	2.612 (6)	158

Symmetry codes: (i) 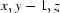; (ii) 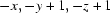.

**Fig. 1.**
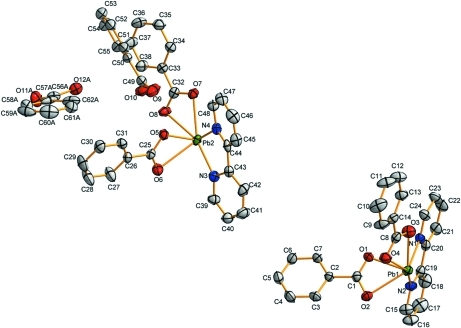
The asymmetric unit of the title complex, showing displacement ellipsoids at the 30% probability level and the atom-labeling scheme. The H atoms and one of the components of the disordered benzoic acid molecule have been omitted for clarity.

**Fig. 2.**
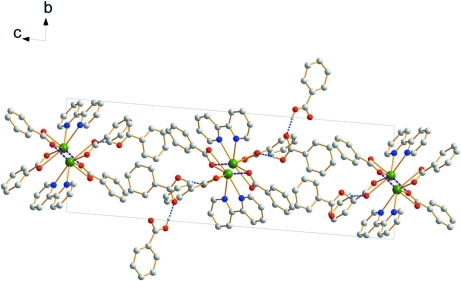
The packing diagram viewed down the a axis; the H atoms not involved in H-bonding and the minor component of the disordered benzoic acid molecule are omitted for clarity. The H-bonds are shown as dashed lines in blue; the weak intermolecular Pb···O interactions are drawn as dashed lines in violet.

## supplementary crystallographic information

### Comment

Complexes containing Pb(II) ion have recently attracted considerable interest not only because of the variety of their architectures, but also because of their potential applications, especially in environmental protection and in systems with different biological properties (Fan & Zhu, 2006; Hamilton *et al.*, 2004; Alvarado *et al.*, 2005). As an important family of bidentate O-donor ligands, aromatic carboxylates have been extensively employed in the preparation of metal complexes of various structural topologies (Wang *et al.*, 2006; Masaoka *et al.*, 2001).

The asymmetric unit of the crystal of the title complex, [Pb(C_7_H_5_O_2_)_2_(C_10_H_8_N_2_)].(C_7_H_6_O_2_), contains two molecules of complex, as well as two independent benzoic solvate molecules (Fig.1), one of which is disordered over two positions with almost equal occupancies. Each Pb^II^ atom is hexacoordinated and chelated by four carboxylate O atoms from two benzoic acid and two N atoms from 2,2'-bipyridine ligand. In both complex molecules the O atoms of one of the carboxylate ligands (O3 and O4 in the first molecule; O7 and O8 in the second molecule) are almost coplanar with the N atoms of the bipy-ligand (N1 and N2 in the first and N3 and N4 in the second molecule). Therefore, if we consider, that the second carboxylate ligand occupies just one coordination site, then coordination environments of Pb1 and Pb2 atoms may be described as pseudo-square-pyramidal.

The weak intermolecular interactions Pb1···O2^i^ [3.359 (3) Å, ^i^ -*x*, 1 - *y*, 2 - *z*] and Pb2···O7^ii^ [3.046 (3) Å, ^ii^ 1 - *x*, 1 - *y*,1 - *z*] link the molecules of complex into centrosymmetric dimers (Fig.2). The H-bonds involving carboxylic H atoms of solvate benzoic acid molecules and metal coordinated carboxylate O atoms (Table 1), link molecules of the complex and benzoic acid solvates into insular aggregates.

### Experimental

A mixture of Pb(CH_3_COO)_2_.3H_2_O (0.199 g, 0.52 mmol), benzoic acid (0.102 g, 0.84 mmol), 2,2'-bipyridine (0.033 g, 0.21 mmol) and distilled water (10 ml) was sealed in a 25 ml Teflon-lined stainless autoclave. The mixture was heated at 393 K for 6 days to give the colorless crystals suitable for X-ray diffraction analysis.

### Refinement

All H atoms bound to C atoms were placed in calculated positions and treated in a riding-model approximation, with C —H = 0.93 Å and *U*_iso_ (H) = 1.2 *U*_eq_(C). The positions of carboxylic H atoms were located in the difference Fourier maps and included in the refinement in riding motion approximation with idealized distance of O—H = 0.85 Å and *U*_iso_ (H) = 1.2 *U*_eq_(O). One of the the benzoic acid molecules [C56—C57—C58—C59—C60—C61—C62—O11—O12—H12] showed high thermal parameters and was subsequently represented as disordered over two sites, with refined occupancies of 0.496 (5) and 0.504 (5). The displacement parameters for the corresponding non-hydrogen atoms in the disordered phenyl rings were set equal (six EADP instructions; see, Sheldrick, 2008). The highest residual peak of 1.17 e Å^-3^ is located at the distance of 0.89 Å from Pb1; the deepest hole -1.13 e Å^-3^ is at 0.65 Å from the same atom.

### Crystal data

**Table:**

[Pb(C_7_H_5_O_2_)_2_(C_10_H_8_N_2_)]·C_7_H_6_O_2_	*Z* = 4
*M**_r_* = 727.71	*F*(000) = 1416
Triclinic, *P*1	*D*_x_ = 1.763 Mg m^−^^3^
Hall symbol: -P 1	Mo *K*α radiation, λ = 0.71073 Å
*a* = 9.6298 (2) Å	Cell parameters from 9902 reflections
*b* = 10.4264 (2) Å	θ = 0.7–29.0°
*c* = 28.7365 (5) Å	µ = 6.2 mm^−^^1^
α = 84.843 (1)°	*T* = 296 K
β = 88.128 (1)°	Prism, colorless
γ = 72.619 (1)°	0.24 × 0.19 × 0.15 mm
*V* = 2742.32 (9) Å^3^	

### Data collection

**Table:**

Bruker APEXII CCD area-detector diffractometer	14302 independent reflections
Radiation source: fine-focus sealed tube	10344 reflections with *I* > 2σ(*I*)
graphite	*R*_int_ = 0.039
φ and ω scans	θ_max_ = 29.0°, θ_min_ = 0.7°
Absorption correction: multi-scan (*SADABS*; Bruker, 2007)	*h* = −13→13
*T*_min_ = 0.253, *T*_max_ = 0.395	*k* = −14→14
49796 measured reflections	*l* = −39→38

### Refinement

**Table:**

Refinement on *F*^2^	Primary atom site location: structure-invariant direct methods
Least-squares matrix: full	Secondary atom site location: difference Fourier map
*R*[*F*^2^ > 2σ(*F*^2^)] = 0.035	Hydrogen site location: inferred from neighbouring sites
*wR*(*F*^2^) = 0.081	H-atom parameters constrained
*S* = 1.01	*w* = 1/[σ^2^(*F*_o_^2^) + (0.0359*P*)^2^ + 1.7335*P*] where *P* = (*F*_o_^2^ + 2*F*_c_^2^)/3
14302 reflections	(Δ/σ)_max_ = 0.001
773 parameters	Δρ_max_ = 1.17 e Å^−^^3^
0 restraints	Δρ_min_ = −1.13 e Å^−^^3^

### Fractional atomic coordinates and isotropic or equivalent isotropic displacement parameters (Å^2^)

**Table:**

	*x*	*y*	*z*	*U*_iso_*/*U*_eq_	Occ. (<1)
Pb1	0.277299 (17)	0.440644 (16)	0.990310 (6)	0.04756 (5)	
O1	0.2560 (3)	0.3122 (3)	0.92887 (10)	0.0547 (8)	
O2	0.0401 (4)	0.4189 (4)	0.95610 (12)	0.0651 (9)	
O3	0.4510 (4)	0.5195 (4)	0.93842 (13)	0.0708 (10)	
O4	0.2300 (4)	0.6359 (4)	0.91565 (12)	0.0686 (9)	
N1	0.5126 (4)	0.2401 (4)	1.00026 (13)	0.0481 (8)	
N2	0.2611 (4)	0.2258 (4)	1.04054 (13)	0.0550 (9)	
C1	0.1188 (5)	0.3474 (5)	0.92752 (15)	0.0498 (10)	
C2	0.0484 (4)	0.2956 (5)	0.88972 (14)	0.0472 (10)	
C3	−0.0994 (5)	0.3513 (6)	0.8821 (2)	0.0701 (15)	
H3A	−0.1541	0.4177	0.9004	0.084*	
C4	−0.1656 (6)	0.3084 (7)	0.8472 (2)	0.0832 (18)	
H4A	−0.2649	0.3464	0.8422	0.100*	
C5	−0.0878 (6)	0.2115 (6)	0.8203 (2)	0.0745 (15)	
H5A	−0.1329	0.1851	0.7963	0.089*	
C6	0.0567 (6)	0.1529 (6)	0.82847 (18)	0.0720 (15)	
H6A	0.1093	0.0828	0.8112	0.086*	
C7	0.1257 (5)	0.1975 (5)	0.86266 (16)	0.0588 (12)	
H7A	0.2253	0.1601	0.8671	0.071*	
C8	0.3649 (5)	0.6109 (5)	0.91275 (16)	0.0550 (11)	
C9	0.3456 (6)	0.7990 (6)	0.85076 (19)	0.0774 (17)	
H9A	0.2458	0.8298	0.8561	0.093*	
C10	0.4109 (8)	0.8652 (7)	0.8160 (2)	0.101 (2)	
H10A	0.3540	0.9397	0.7980	0.121*	
C11	0.5556 (8)	0.8217 (7)	0.8087 (3)	0.104 (2)	
H11A	0.5980	0.8650	0.7852	0.125*	
C12	0.6389 (7)	0.7157 (7)	0.8353 (3)	0.093 (2)	
H12A	0.7392	0.6876	0.8306	0.112*	
C13	0.5777 (6)	0.6490 (6)	0.86907 (19)	0.0689 (14)	
H13A	0.6367	0.5758	0.8871	0.083*	
C14	0.4301 (5)	0.6884 (5)	0.87676 (15)	0.0524 (11)	
C15	0.1347 (6)	0.2245 (6)	1.0599 (2)	0.0748 (15)	
H15A	0.0525	0.2960	1.0518	0.090*	
C16	0.1199 (8)	0.1232 (7)	1.0910 (2)	0.0899 (19)	
H16A	0.0292	0.1248	1.1036	0.108*	
C17	0.2395 (9)	0.0198 (7)	1.1034 (2)	0.098 (2)	
H17A	0.2321	−0.0506	1.1247	0.118*	
C18	0.3724 (7)	0.0200 (6)	1.08416 (19)	0.0774 (16)	
H18A	0.4558	−0.0499	1.0924	0.093*	
C19	0.3799 (5)	0.1252 (5)	1.05259 (15)	0.0522 (11)	
C20	0.5193 (5)	0.1345 (5)	1.03072 (15)	0.0492 (10)	
C21	0.6504 (6)	0.0360 (5)	1.04115 (19)	0.0673 (14)	
H21A	0.6527	−0.0403	1.0609	0.081*	
C22	0.7757 (6)	0.0531 (7)	1.0220 (2)	0.0832 (18)	
H22A	0.8650	−0.0083	1.0304	0.100*	
C23	0.7700 (5)	0.1604 (6)	0.9904 (2)	0.0806 (17)	
H23A	0.8542	0.1715	0.9761	0.097*	
C24	0.6360 (5)	0.2517 (5)	0.98016 (19)	0.0618 (13)	
H24A	0.6312	0.3245	0.9584	0.074*	
Pb2	0.260139 (17)	0.547049 (16)	0.492170 (5)	0.04398 (5)	
O5	0.1638 (3)	0.6596 (3)	0.41827 (10)	0.0517 (7)	
O6	−0.0072 (4)	0.5925 (4)	0.45613 (11)	0.0684 (10)	
O7	0.4867 (3)	0.4786 (3)	0.44324 (10)	0.0539 (8)	
O8	0.3453 (4)	0.3496 (3)	0.43410 (12)	0.0654 (9)	
N3	0.0919 (4)	0.7500 (4)	0.53414 (13)	0.0516 (9)	
N4	0.3238 (4)	0.7697 (4)	0.48246 (12)	0.0496 (9)	
C25	0.0347 (5)	0.6481 (4)	0.42089 (14)	0.0462 (10)	
C26	−0.0617 (5)	0.7019 (4)	0.38005 (15)	0.0468 (10)	
C27	−0.1856 (6)	0.6646 (7)	0.3762 (2)	0.087 (2)	
H27A	−0.2102	0.6087	0.4000	0.105*	
C28	−0.2740 (7)	0.7079 (8)	0.3380 (3)	0.109 (3)	
H28A	−0.3568	0.6803	0.3359	0.131*	
C29	−0.2417 (7)	0.7903 (7)	0.3033 (2)	0.094 (2)	
H29A	−0.3013	0.8188	0.2772	0.113*	
C30	−0.1203 (6)	0.8320 (6)	0.30662 (18)	0.0682 (14)	
H30A	−0.0987	0.8904	0.2831	0.082*	
C31	−0.0309 (5)	0.7874 (5)	0.34468 (16)	0.0505 (10)	
H31A	0.0516	0.8155	0.3466	0.061*	
C32	0.4552 (5)	0.3858 (4)	0.42343 (15)	0.0476 (10)	
C33	0.5526 (4)	0.3229 (4)	0.38502 (14)	0.0443 (9)	
C34	0.6673 (5)	0.3672 (5)	0.36900 (17)	0.0588 (12)	
H34A	0.6875	0.4360	0.3834	0.071*	
C35	0.7531 (6)	0.3129 (6)	0.3324 (2)	0.0751 (16)	
H35A	0.8297	0.3455	0.3220	0.090*	
C36	0.7259 (7)	0.2110 (7)	0.3112 (2)	0.0877 (19)	
H36A	0.7837	0.1736	0.2864	0.105*	
C37	0.6126 (8)	0.1644 (7)	0.3268 (2)	0.096 (2)	
H37A	0.5944	0.0941	0.3127	0.116*	
C38	0.5253 (6)	0.2207 (5)	0.3631 (2)	0.0702 (15)	
H38A	0.4474	0.1895	0.3729	0.084*	
C39	−0.0144 (5)	0.7296 (5)	0.56216 (18)	0.0634 (13)	
H39A	−0.0436	0.6535	0.5592	0.076*	
C40	−0.0815 (6)	0.8135 (6)	0.5945 (2)	0.0759 (16)	
H40A	−0.1560	0.7963	0.6130	0.091*	
C41	−0.0370 (7)	0.9251 (7)	0.5994 (2)	0.0896 (19)	
H41A	−0.0800	0.9842	0.6217	0.108*	
C42	0.0709 (6)	0.9484 (6)	0.57104 (19)	0.0719 (15)	
H42A	0.1008	1.0244	0.5736	0.086*	
C43	0.1358 (5)	0.8587 (4)	0.53846 (15)	0.0489 (10)	
C44	0.2496 (5)	0.8770 (4)	0.50499 (15)	0.0477 (10)	
C45	0.2771 (6)	0.9997 (5)	0.49649 (19)	0.0656 (14)	
H45A	0.2279	1.0718	0.5134	0.079*	
C46	0.3775 (6)	1.0149 (6)	0.4629 (2)	0.0761 (16)	
H46A	0.3948	1.0978	0.4562	0.091*	
C47	0.4508 (6)	0.9065 (6)	0.43973 (19)	0.0704 (15)	
H47A	0.5203	0.9143	0.4173	0.084*	
C48	0.4218 (5)	0.7836 (5)	0.44951 (17)	0.0595 (12)	
H48A	0.4708	0.7104	0.4330	0.071*	
O9	0.3064 (6)	0.8312 (6)	0.32918 (16)	0.1072 (16)	
O10	0.3206 (4)	0.6176 (4)	0.33971 (12)	0.0809 (11)	
H10	0.2758	0.6364	0.3653	0.097*	
C49	0.3429 (5)	0.7208 (8)	0.31470 (19)	0.0713 (16)	
C50	0.4195 (5)	0.6882 (7)	0.27012 (16)	0.0645 (15)	
C51	0.4360 (5)	0.7919 (7)	0.23990 (18)	0.0747 (16)	
H51A	0.3978	0.8807	0.2472	0.090*	
C52	0.5129 (6)	0.7617 (9)	0.1968 (2)	0.088 (2)	
H52A	0.5251	0.8313	0.1760	0.105*	
C53	0.5679 (7)	0.6322 (10)	0.1863 (2)	0.098 (2)	
H53A	0.6174	0.6121	0.1583	0.117*	
C54	0.5493 (7)	0.5302 (9)	0.2178 (2)	0.095 (2)	
H54A	0.5890	0.4411	0.2109	0.113*	
C55	0.4748 (6)	0.5561 (7)	0.25873 (19)	0.0782 (18)	
H55A	0.4612	0.4857	0.2788	0.094*	
O11A	0.1002 (9)	0.0133 (8)	0.2570 (3)	0.089 (3)	0.496 (5)
O12A	0.2529 (7)	0.0710 (6)	0.3016 (2)	0.0624 (19)	0.496 (5)
H12C	0.2823	−0.0120	0.3113	0.075*	0.496 (5)
C56A	0.1453 (11)	0.0975 (10)	0.2712 (4)	0.054 (2)	0.496 (5)
C57A	0.0816 (7)	0.2391 (6)	0.24973 (19)	0.081 (6)	0.496 (5)
C58A	−0.0179 (5)	0.2866 (5)	0.21357 (15)	0.089 (6)	0.496 (5)
H58A	−0.0459	0.2262	0.1969	0.107*	0.496 (5)
C59A	−0.0754 (5)	0.4246 (5)	0.2023 (2)	0.101 (5)	0.496 (5)
H59A	−0.1419	0.4564	0.1781	0.121*	0.496 (5)
C60A	−0.0334 (8)	0.5150 (6)	0.2273 (3)	0.098 (5)	0.496 (5)
H60A	−0.0719	0.6073	0.2197	0.117*	0.496 (5)
C61A	0.0661 (9)	0.4674 (8)	0.2634 (2)	0.097 (6)	0.496 (5)
H61A	0.0941	0.5279	0.2801	0.116*	0.496 (5)
C62A	0.1236 (8)	0.3294 (9)	0.2747 (2)	0.088 (5)	0.496 (5)
H62A	0.1901	0.2976	0.2989	0.105*	0.496 (5)
O11B	−0.0444 (8)	0.1612 (5)	0.1905 (2)	0.082 (2)	0.504 (5)
O12B	−0.0935 (6)	0.3742 (6)	0.16037 (18)	0.066 (2)	0.504 (5)
H12B	−0.1336	0.3492	0.1384	0.079*	0.504 (5)
C56B	−0.0404 (13)	0.2721 (13)	0.1906 (4)	0.057 (3)	0.504 (5)
C57B	0.0307 (7)	0.3092 (8)	0.2309 (2)	0.045 (3)	0.504 (5)
C58B	0.0251 (9)	0.4409 (7)	0.2377 (3)	0.066 (3)	0.504 (5)
H58B	−0.0326	0.5123	0.2186	0.079*	0.504 (5)
C59B	0.1058 (10)	0.4660 (7)	0.2732 (3)	0.079 (5)	0.504 (5)
H59B	0.1020	0.5542	0.2778	0.095*	0.504 (5)
C60B	0.1921 (9)	0.3594 (9)	0.3018 (3)	0.082 (4)	0.504 (5)
H60B	0.2461	0.3762	0.3255	0.098*	0.504 (5)
C61B	0.1977 (8)	0.2276 (8)	0.2950 (2)	0.083 (4)	0.504 (5)
H61B	0.2555	0.1563	0.3141	0.099*	0.504 (5)
C62B	0.1171 (8)	0.2025 (6)	0.2595 (3)	0.057 (4)	0.504 (5)
H62B	0.1208	0.1144	0.2549	0.068*	0.504 (5)

### Atomic displacement parameters (Å^2^)

**Table:**

	*U*^11^	*U*^22^	*U*^33^	*U*^12^	*U*^13^	*U*^23^
Pb1	0.05027 (10)	0.04123 (10)	0.04718 (10)	−0.00703 (7)	−0.00101 (7)	−0.00521 (7)
O1	0.0420 (16)	0.064 (2)	0.0543 (18)	−0.0066 (14)	−0.0052 (13)	−0.0125 (16)
O2	0.0580 (19)	0.072 (2)	0.061 (2)	−0.0073 (17)	0.0029 (16)	−0.0239 (18)
O3	0.070 (2)	0.059 (2)	0.071 (2)	−0.0065 (18)	0.0017 (18)	0.0121 (19)
O4	0.059 (2)	0.079 (3)	0.063 (2)	−0.0167 (18)	0.0036 (16)	0.0039 (19)
N1	0.0443 (19)	0.042 (2)	0.056 (2)	−0.0074 (16)	−0.0069 (16)	−0.0083 (17)
N2	0.051 (2)	0.051 (2)	0.056 (2)	−0.0076 (18)	0.0036 (17)	−0.0005 (18)
C1	0.051 (3)	0.052 (3)	0.045 (2)	−0.012 (2)	−0.0015 (19)	0.000 (2)
C2	0.043 (2)	0.055 (3)	0.040 (2)	−0.010 (2)	−0.0002 (17)	−0.002 (2)
C3	0.041 (2)	0.084 (4)	0.080 (4)	−0.005 (2)	−0.004 (2)	−0.021 (3)
C4	0.049 (3)	0.096 (5)	0.102 (5)	−0.012 (3)	−0.021 (3)	−0.020 (4)
C5	0.067 (3)	0.089 (4)	0.074 (4)	−0.030 (3)	−0.021 (3)	−0.011 (3)
C6	0.077 (4)	0.078 (4)	0.053 (3)	−0.006 (3)	−0.005 (2)	−0.021 (3)
C7	0.046 (2)	0.071 (3)	0.052 (3)	−0.006 (2)	−0.005 (2)	−0.009 (2)
C8	0.065 (3)	0.051 (3)	0.043 (2)	−0.006 (2)	−0.001 (2)	−0.009 (2)
C9	0.070 (3)	0.067 (4)	0.070 (3)	0.010 (3)	0.017 (3)	0.010 (3)
C10	0.124 (6)	0.071 (4)	0.082 (4)	0.000 (4)	0.021 (4)	0.022 (3)
C11	0.115 (6)	0.077 (5)	0.107 (5)	−0.019 (4)	0.056 (4)	0.003 (4)
C12	0.076 (4)	0.093 (5)	0.112 (5)	−0.029 (4)	0.034 (4)	−0.012 (4)
C13	0.063 (3)	0.064 (3)	0.073 (3)	−0.010 (3)	0.004 (3)	−0.006 (3)
C14	0.058 (3)	0.050 (3)	0.045 (2)	−0.010 (2)	0.007 (2)	−0.009 (2)
C15	0.069 (3)	0.073 (4)	0.076 (4)	−0.016 (3)	0.017 (3)	−0.003 (3)
C16	0.094 (5)	0.088 (5)	0.090 (4)	−0.035 (4)	0.025 (4)	0.002 (4)
C17	0.127 (6)	0.081 (5)	0.088 (5)	−0.041 (4)	0.010 (4)	0.022 (4)
C18	0.094 (4)	0.062 (4)	0.070 (4)	−0.018 (3)	−0.010 (3)	0.015 (3)
C19	0.065 (3)	0.044 (3)	0.045 (2)	−0.011 (2)	−0.006 (2)	−0.006 (2)
C20	0.052 (2)	0.044 (3)	0.047 (2)	−0.0047 (19)	−0.0122 (19)	−0.010 (2)
C21	0.065 (3)	0.057 (3)	0.067 (3)	0.002 (2)	−0.013 (3)	0.003 (3)
C22	0.056 (3)	0.082 (5)	0.092 (4)	0.010 (3)	−0.018 (3)	−0.007 (4)
C23	0.043 (3)	0.078 (4)	0.113 (5)	−0.005 (3)	−0.001 (3)	−0.015 (4)
C24	0.047 (3)	0.058 (3)	0.080 (3)	−0.014 (2)	−0.001 (2)	−0.010 (3)
Pb2	0.05568 (10)	0.04077 (10)	0.03725 (9)	−0.01737 (7)	−0.00074 (7)	−0.00191 (7)
O5	0.0558 (18)	0.061 (2)	0.0414 (16)	−0.0237 (15)	−0.0062 (13)	0.0025 (14)
O6	0.078 (2)	0.088 (3)	0.0513 (19)	−0.048 (2)	−0.0061 (16)	0.0106 (18)
O7	0.0624 (19)	0.052 (2)	0.0499 (17)	−0.0199 (15)	0.0022 (14)	−0.0101 (15)
O8	0.069 (2)	0.066 (2)	0.073 (2)	−0.0363 (18)	0.0264 (17)	−0.0249 (18)
N3	0.056 (2)	0.041 (2)	0.060 (2)	−0.0181 (17)	0.0005 (18)	−0.0059 (18)
N4	0.059 (2)	0.048 (2)	0.046 (2)	−0.0217 (18)	−0.0087 (17)	0.0001 (17)
C25	0.056 (3)	0.046 (3)	0.039 (2)	−0.020 (2)	−0.0021 (18)	−0.0071 (19)
C26	0.051 (2)	0.046 (3)	0.046 (2)	−0.017 (2)	−0.0032 (18)	−0.008 (2)
C27	0.081 (4)	0.114 (5)	0.082 (4)	−0.060 (4)	−0.029 (3)	0.028 (4)
C28	0.088 (4)	0.140 (7)	0.117 (6)	−0.069 (5)	−0.050 (4)	0.030 (5)
C29	0.088 (4)	0.101 (5)	0.092 (5)	−0.032 (4)	−0.046 (4)	0.016 (4)
C30	0.074 (3)	0.065 (3)	0.058 (3)	−0.012 (3)	−0.011 (3)	0.010 (3)
C31	0.050 (2)	0.046 (3)	0.053 (3)	−0.012 (2)	−0.0044 (19)	−0.004 (2)
C32	0.057 (3)	0.039 (2)	0.046 (2)	−0.013 (2)	−0.0019 (19)	−0.002 (2)
C33	0.049 (2)	0.038 (2)	0.045 (2)	−0.0111 (18)	−0.0006 (18)	0.0008 (18)
C34	0.055 (3)	0.066 (3)	0.059 (3)	−0.024 (2)	0.002 (2)	−0.008 (2)
C35	0.061 (3)	0.089 (4)	0.075 (4)	−0.025 (3)	0.015 (3)	−0.004 (3)
C36	0.083 (4)	0.091 (5)	0.084 (4)	−0.017 (4)	0.033 (3)	−0.025 (4)
C37	0.114 (5)	0.088 (5)	0.101 (5)	−0.042 (4)	0.037 (4)	−0.055 (4)
C38	0.074 (3)	0.059 (3)	0.087 (4)	−0.031 (3)	0.021 (3)	−0.028 (3)
C39	0.061 (3)	0.061 (3)	0.070 (3)	−0.022 (3)	0.006 (2)	−0.005 (3)
C40	0.067 (3)	0.085 (4)	0.078 (4)	−0.026 (3)	0.019 (3)	−0.017 (3)
C41	0.095 (4)	0.091 (5)	0.088 (4)	−0.028 (4)	0.029 (4)	−0.040 (4)
C42	0.094 (4)	0.056 (3)	0.072 (4)	−0.027 (3)	0.006 (3)	−0.025 (3)
C43	0.054 (2)	0.043 (3)	0.049 (2)	−0.013 (2)	−0.0095 (19)	−0.004 (2)
C44	0.057 (3)	0.037 (2)	0.051 (2)	−0.018 (2)	−0.019 (2)	0.003 (2)
C45	0.078 (3)	0.044 (3)	0.078 (4)	−0.023 (3)	−0.008 (3)	−0.004 (3)
C46	0.082 (4)	0.048 (3)	0.104 (5)	−0.032 (3)	−0.012 (3)	0.010 (3)
C47	0.079 (4)	0.071 (4)	0.071 (3)	−0.043 (3)	−0.001 (3)	0.012 (3)
C48	0.066 (3)	0.063 (3)	0.055 (3)	−0.028 (3)	0.000 (2)	0.002 (2)
O9	0.135 (4)	0.127 (4)	0.089 (3)	−0.079 (4)	0.048 (3)	−0.040 (3)
O10	0.079 (2)	0.096 (3)	0.054 (2)	−0.010 (2)	0.0122 (18)	0.006 (2)
C49	0.050 (3)	0.115 (5)	0.054 (3)	−0.033 (3)	−0.001 (2)	−0.011 (3)
C50	0.046 (3)	0.110 (5)	0.043 (3)	−0.030 (3)	−0.001 (2)	−0.008 (3)
C51	0.056 (3)	0.115 (5)	0.060 (3)	−0.037 (3)	−0.002 (2)	−0.003 (3)
C52	0.066 (4)	0.144 (7)	0.062 (4)	−0.048 (4)	−0.004 (3)	0.002 (4)
C53	0.076 (4)	0.169 (8)	0.057 (4)	−0.045 (5)	0.006 (3)	−0.025 (5)
C54	0.090 (5)	0.129 (6)	0.067 (4)	−0.031 (4)	0.008 (3)	−0.033 (4)
C55	0.067 (4)	0.104 (5)	0.063 (4)	−0.019 (3)	0.004 (3)	−0.026 (4)
O11A	0.100 (6)	0.061 (5)	0.117 (7)	−0.035 (5)	−0.016 (5)	−0.022 (5)
O12A	0.074 (5)	0.040 (4)	0.070 (5)	−0.013 (3)	0.004 (4)	−0.002 (3)
C56A	0.055 (6)	0.041 (6)	0.064 (6)	−0.013 (5)	0.015 (5)	−0.002 (5)
C57A	0.096 (11)	0.049 (8)	0.113 (15)	−0.042 (9)	0.071 (11)	−0.041 (10)
C58A	0.060 (9)	0.136 (17)	0.081 (14)	−0.040 (9)	0.005 (9)	−0.026 (12)
C59A	0.066 (8)	0.092 (11)	0.121 (13)	−0.002 (7)	0.015 (8)	0.033 (10)
C60A	0.097 (11)	0.051 (8)	0.135 (14)	−0.015 (7)	0.026 (9)	0.016 (9)
C61A	0.080 (9)	0.148 (19)	0.076 (11)	−0.053 (11)	0.004 (9)	−0.011 (11)
C62A	0.067 (7)	0.085 (10)	0.076 (9)	0.013 (7)	0.021 (6)	0.042 (8)
O11B	0.098 (6)	0.065 (5)	0.090 (6)	−0.032 (4)	−0.020 (4)	−0.008 (4)
O12B	0.076 (5)	0.063 (5)	0.061 (4)	−0.022 (4)	−0.016 (3)	−0.002 (4)
C56B	0.055 (6)	0.057 (7)	0.064 (8)	−0.019 (5)	0.005 (5)	−0.018 (6)
C57B	0.055 (7)	0.030 (5)	0.041 (6)	0.000 (4)	0.007 (4)	0.005 (4)
C58B	0.068 (8)	0.058 (8)	0.061 (7)	−0.009 (6)	−0.001 (6)	0.008 (6)
C59B	0.099 (10)	0.052 (8)	0.103 (11)	−0.038 (7)	−0.012 (8)	−0.031 (7)
C60B	0.092 (9)	0.088 (10)	0.069 (8)	−0.034 (8)	−0.007 (6)	−0.008 (7)
C61B	0.068 (7)	0.102 (11)	0.066 (7)	−0.010 (7)	−0.002 (5)	0.006 (7)
C62B	0.054 (6)	0.109 (13)	0.022 (4)	−0.048 (7)	−0.005 (4)	−0.002 (6)

### Geometric parameters (Å, °)

**Table:**

Pb1—O1	2.359 (3)	C29—H29A	0.9300
Pb1—O2	2.594 (3)	C30—C31	1.373 (6)
Pb1—O3	2.477 (4)	C30—H30A	0.9300
Pb1—O4	2.771 (4)	C31—H31A	0.9300
Pb1—N1	2.587 (3)	C32—C33	1.491 (6)
Pb1—N2	2.596 (4)	C33—C34	1.370 (6)
Pb1—O2^i^	3.359 (3)	C33—C38	1.378 (6)
O1—C1	1.262 (5)	C34—C35	1.372 (7)
O2—C1	1.239 (5)	C34—H34A	0.9300
O3—C8	1.258 (5)	C35—C36	1.365 (8)
O4—C8	1.247 (6)	C35—H35A	0.9300
N1—C20	1.331 (6)	C36—C37	1.368 (8)
N1—C24	1.338 (6)	C36—H36A	0.9300
N2—C15	1.325 (6)	C37—C38	1.379 (7)
N2—C19	1.331 (5)	C37—H37A	0.9300
C1—C2	1.518 (6)	C38—H38A	0.9300
C2—C7	1.362 (6)	C39—C40	1.347 (7)
C2—C3	1.384 (6)	C39—H39A	0.9300
C3—C4	1.379 (7)	C40—C41	1.374 (8)
C3—H3A	0.9300	C40—H40A	0.9300
C4—C5	1.353 (8)	C41—C42	1.365 (8)
C4—H4A	0.9300	C41—H41A	0.9300
C5—C6	1.361 (7)	C42—C43	1.383 (7)
C5—H5A	0.9300	C42—H42A	0.9300
C6—C7	1.390 (7)	C43—C44	1.478 (6)
C6—H6A	0.9300	C44—C45	1.381 (6)
C7—H7A	0.9300	C45—C46	1.377 (8)
C8—C14	1.493 (6)	C45—H45A	0.9300
C9—C14	1.369 (7)	C46—C47	1.359 (8)
C9—C10	1.401 (8)	C46—H46A	0.9300
C9—H9A	0.9300	C47—C48	1.394 (7)
C10—C11	1.346 (9)	C47—H47A	0.9300
C10—H10A	0.9300	C48—H48A	0.9300
C11—C12	1.344 (9)	O9—C49	1.208 (7)
C11—H11A	0.9300	O10—C49	1.307 (7)
C12—C13	1.364 (8)	O10—H10	0.8500
C12—H12A	0.9300	C49—C50	1.472 (7)
C13—C14	1.373 (7)	C50—C51	1.368 (8)
C13—H13A	0.9300	C50—C55	1.385 (8)
C15—C16	1.360 (8)	C51—C52	1.432 (8)
C15—H15A	0.9300	C51—H51A	0.9300
C16—C17	1.353 (9)	C52—C53	1.353 (10)
C16—H16A	0.9300	C52—H52A	0.9300
C17—C18	1.377 (9)	C53—C54	1.384 (10)
C17—H17A	0.9300	C53—H53A	0.9300
C18—C19	1.377 (7)	C54—C55	1.363 (8)
C18—H18A	0.9300	C54—H54A	0.9300
C19—C20	1.489 (6)	C55—H55A	0.9300
C20—C21	1.391 (6)	O11A—C56A	1.197 (12)
C21—C22	1.364 (8)	O12A—C56A	1.325 (12)
C21—H21A	0.9300	O12A—H12C	0.8500
C22—C23	1.364 (8)	C56A—C57A	1.501 (11)
C22—H22A	0.9300	C57A—C58A	1.3900
C23—C24	1.377 (7)	C57A—C62A	1.3900
C23—H23A	0.9300	C58A—C59A	1.3900
C24—H24A	0.9300	C58A—H58A	0.9300
Pb2—O5	2.404 (3)	C59A—C60A	1.3900
Pb2—O6	2.698 (3)	C59A—H59A	0.9300
Pb2—O7	2.509 (3)	C60A—C61A	1.3900
Pb2—O8	2.686 (3)	C60A—H60A	0.9300
Pb2—N3	2.615 (4)	C61A—C62A	1.3900
Pb2—N4	2.562 (4)	C61A—H61A	0.9300
Pb2—O7^ii^	3.046 (3)	C62A—H62A	0.9300
O5—C25	1.283 (5)	O11B—C56B	1.169 (13)
O6—C25	1.238 (5)	O12B—C56B	1.299 (13)
O7—C32	1.279 (5)	O12B—H12B	0.8500
O8—C32	1.247 (5)	C56B—C57B	1.499 (13)
N3—C39	1.336 (6)	C57B—C58B	1.3900
N3—C43	1.339 (5)	C57B—C62B	1.3900
N4—C44	1.343 (6)	C58B—C59B	1.3900
N4—C48	1.344 (6)	C58B—H58B	0.9300
C25—C26	1.482 (6)	C59B—C60B	1.3900
C26—C27	1.372 (6)	C59B—H59B	0.9300
C26—C31	1.375 (6)	C60B—C61B	1.3900
C27—C28	1.370 (8)	C60B—H60B	0.9300
C27—H27A	0.9300	C61B—C62B	1.3900
C28—C29	1.346 (9)	C61B—H61B	0.9300
C28—H28A	0.9300	C62B—H62B	0.9300
C29—C30	1.372 (8)		
			
O1—Pb1—O3	85.77 (12)	O6—C25—C26	121.2 (4)
O1—Pb1—N1	78.51 (10)	O5—C25—C26	117.7 (4)
O3—Pb1—N1	77.71 (11)	C27—C26—C31	117.6 (4)
O1—Pb1—O2	52.47 (10)	C27—C26—C25	119.9 (4)
O3—Pb1—O2	119.86 (12)	C31—C26—C25	122.5 (4)
N1—Pb1—O2	122.69 (11)	C28—C27—C26	121.4 (5)
O1—Pb1—N2	81.89 (12)	C28—C27—H27A	119.3
O3—Pb1—N2	139.91 (11)	C26—C27—H27A	119.3
N1—Pb1—N2	62.50 (12)	C29—C28—C27	120.4 (5)
O2—Pb1—N2	81.03 (12)	C29—C28—H28A	119.8
O1—Pb1—C1	26.42 (11)	C27—C28—H28A	119.8
O3—Pb1—C1	102.85 (13)	C28—C29—C30	119.6 (5)
N1—Pb1—C1	101.75 (12)	C28—C29—H29A	120.2
O2—Pb1—C1	26.10 (11)	C30—C29—H29A	120.2
N2—Pb1—C1	81.72 (13)	C29—C30—C31	120.0 (5)
O1—Pb1—O2^i^	113.58 (9)	C29—C30—H30A	120.0
O3—Pb1—O2^i^	136.89 (10)	C31—C30—H30A	120.0
N1—Pb1—O2^i^	141.64 (11)	C30—C31—C26	120.9 (4)
O2—Pb1—O2^i^	61.44 (11)	C30—C31—H31A	119.5
N2—Pb1—O2^i^	82.59 (10)	C26—C31—H31A	119.5
C1—Pb1—O2^i^	87.48 (11)	O8—C32—O7	122.7 (4)
C1—O1—Pb1	97.3 (3)	O8—C32—C33	119.5 (4)
C1—O2—Pb1	86.8 (3)	O7—C32—C33	117.8 (4)
C8—O3—Pb1	100.9 (3)	C34—C33—C38	117.8 (4)
C20—N1—C24	118.6 (4)	C34—C33—C32	122.0 (4)
C20—N1—Pb1	121.2 (3)	C38—C33—C32	120.2 (4)
C24—N1—Pb1	119.3 (3)	C33—C34—C35	121.9 (5)
C15—N2—C19	119.0 (4)	C33—C34—H34A	119.0
C15—N2—Pb1	118.9 (3)	C35—C34—H34A	119.0
C19—N2—Pb1	121.4 (3)	C36—C35—C34	119.8 (5)
O2—C1—O1	123.2 (4)	C36—C35—H35A	120.1
O2—C1—C2	119.1 (4)	C34—C35—H35A	120.1
O1—C1—C2	117.8 (4)	C35—C36—C37	119.3 (5)
O2—C1—Pb1	67.1 (3)	C35—C36—H36A	120.3
O1—C1—Pb1	56.3 (2)	C37—C36—H36A	120.3
C2—C1—Pb1	173.2 (3)	C36—C37—C38	120.5 (6)
C7—C2—C3	118.8 (4)	C36—C37—H37A	119.7
C7—C2—C1	122.4 (4)	C38—C37—H37A	119.7
C3—C2—C1	118.8 (4)	C33—C38—C37	120.6 (5)
C4—C3—C2	120.0 (5)	C33—C38—H38A	119.7
C4—C3—H3A	120.0	C37—C38—H38A	119.7
C2—C3—H3A	120.0	N3—C39—C40	123.6 (5)
C5—C4—C3	120.8 (5)	N3—C39—H39A	118.2
C5—C4—H4A	119.6	C40—C39—H39A	118.2
C3—C4—H4A	119.6	C39—C40—C41	118.2 (5)
C4—C5—C6	119.8 (5)	C39—C40—H40A	120.9
C4—C5—H5A	120.1	C41—C40—H40A	120.9
C6—C5—H5A	120.1	C42—C41—C40	119.3 (5)
C5—C6—C7	120.1 (5)	C42—C41—H41A	120.3
C5—C6—H6A	120.0	C40—C41—H41A	120.3
C7—C6—H6A	120.0	C41—C42—C43	119.8 (5)
C2—C7—C6	120.5 (4)	C41—C42—H42A	120.1
C2—C7—H7A	119.8	C43—C42—H42A	120.1
C6—C7—H7A	119.8	N3—C43—C42	120.3 (4)
O4—C8—O3	122.7 (5)	N3—C43—C44	115.5 (4)
O4—C8—C14	119.9 (4)	C42—C43—C44	124.2 (4)
O3—C8—C14	117.3 (4)	N4—C44—C45	121.2 (4)
C14—C9—C10	119.3 (5)	N4—C44—C43	117.2 (4)
C14—C9—H9A	120.3	C45—C44—C43	121.6 (4)
C10—C9—H9A	120.3	C46—C45—C44	119.9 (5)
C11—C10—C9	120.4 (6)	C46—C45—H45A	120.1
C11—C10—H10A	119.8	C44—C45—H45A	120.1
C9—C10—H10A	119.8	C47—C46—C45	118.7 (5)
C12—C11—C10	120.1 (6)	C47—C46—H46A	120.7
C12—C11—H11A	119.9	C45—C46—H46A	120.7
C10—C11—H11A	119.9	C46—C47—C48	120.1 (5)
C11—C12—C13	120.5 (6)	C46—C47—H47A	120.0
C11—C12—H12A	119.7	C48—C47—H47A	120.0
C13—C12—H12A	119.7	N4—C48—C47	120.8 (5)
C12—C13—C14	120.9 (6)	N4—C48—H48A	119.6
C12—C13—H13A	119.5	C47—C48—H48A	119.6
C14—C13—H13A	119.5	C49—O10—H10	114.6
C9—C14—C13	118.7 (5)	O9—C49—O10	120.6 (5)
C9—C14—C8	121.4 (4)	O9—C49—C50	124.8 (6)
C13—C14—C8	120.0 (4)	O10—C49—C50	114.6 (6)
N2—C15—C16	122.8 (6)	C51—C50—C55	120.2 (5)
N2—C15—H15A	118.6	C51—C50—C49	118.4 (6)
C16—C15—H15A	118.6	C55—C50—C49	121.3 (6)
C17—C16—C15	118.8 (6)	C50—C51—C52	119.1 (6)
C17—C16—H16A	120.6	C50—C51—H51A	120.4
C15—C16—H16A	120.6	C52—C51—H51A	120.4
C16—C17—C18	119.2 (6)	C53—C52—C51	120.2 (7)
C16—C17—H17A	120.4	C53—C52—H52A	119.9
C18—C17—H17A	120.4	C51—C52—H52A	119.9
C19—C18—C17	119.1 (6)	C52—C53—C54	119.0 (6)
C19—C18—H18A	120.4	C52—C53—H53A	120.5
C17—C18—H18A	120.4	C54—C53—H53A	120.5
N2—C19—C18	120.9 (5)	C55—C54—C53	122.1 (7)
N2—C19—C20	116.5 (4)	C55—C54—H54A	119.0
C18—C19—C20	122.6 (5)	C53—C54—H54A	119.0
N1—C20—C21	121.4 (5)	C54—C55—C50	119.4 (7)
N1—C20—C19	117.1 (4)	C54—C55—H55A	120.3
C21—C20—C19	121.4 (5)	C50—C55—H55A	120.3
C22—C21—C20	118.9 (5)	C56A—O12A—H12C	112.4
C22—C21—H21A	120.5	O11A—C56A—O12A	123.9 (10)
C20—C21—H21A	120.5	O11A—C56A—C57A	116.3 (10)
C21—C22—C23	120.0 (5)	O12A—C56A—C57A	119.6 (8)
C21—C22—H22A	120.0	C58A—C57A—C62A	120.0
C23—C22—H22A	120.0	C58A—C57A—C56A	129.3 (5)
C22—C23—C24	118.2 (5)	C62A—C57A—C56A	110.4 (5)
C22—C23—H23A	120.9	C59A—C58A—C57A	120.0
C24—C23—H23A	120.9	C59A—C58A—H58A	120.0
N1—C24—C23	122.8 (5)	C57A—C58A—H58A	120.0
N1—C24—H24A	118.6	C60A—C59A—C58A	120.0
C23—C24—H24A	118.6	C60A—C59A—H59A	120.0
O5—Pb2—O7	80.77 (10)	C58A—C59A—H59A	120.0
O5—Pb2—N4	72.88 (10)	C59A—C60A—C61A	120.0
O7—Pb2—N4	79.87 (11)	C59A—C60A—H60A	120.0
O5—Pb2—N3	89.37 (11)	C61A—C60A—H60A	120.0
O7—Pb2—N3	142.43 (11)	C62A—C61A—C60A	120.0
N4—Pb2—N3	62.61 (12)	C62A—C61A—H61A	120.0
O5—Pb2—O8	77.15 (11)	C60A—C61A—H61A	120.0
O7—Pb2—O8	50.36 (10)	C61A—C62A—C57A	120.0
N4—Pb2—O8	125.09 (11)	C61A—C62A—H62A	120.0
N3—Pb2—O8	160.03 (11)	C57A—C62A—H62A	120.0
O5—Pb2—O6	50.57 (9)	C56B—O12B—H12B	109.5
O7—Pb2—O6	122.01 (10)	O11B—C56B—O12B	128.9 (10)
N4—Pb2—O6	107.79 (11)	O11B—C56B—C57B	118.7 (11)
N3—Pb2—O6	74.58 (11)	O12B—C56B—C57B	112.5 (10)
O8—Pb2—O6	85.46 (11)	C58B—C57B—C62B	120.0
O5—Pb2—O7^ii^	139.48 (9)	C58B—C57B—C56B	123.4 (8)
O7—Pb2—O7^ii^	73.74 (10)	C62B—C57B—C56B	116.3 (8)
N4—Pb2—O7^ii^	72.03 (10)	C57B—C58B—C59B	120.0
N3—Pb2—O7^ii^	92.05 (10)	C57B—C58B—H58B	120.0
O8—Pb2—O7^ii^	107.79 (10)	C59B—C58B—H58B	120.0
O6—Pb2—O7^ii^	164.22 (10)	C60B—C59B—C58B	120.0
C25—O5—Pb2	100.5 (2)	C60B—C59B—H59B	120.0
C25—O6—Pb2	87.8 (3)	C58B—C59B—H59B	120.0
C32—O7—Pb2	97.1 (3)	C61B—C60B—C59B	120.0
C32—O8—Pb2	89.6 (3)	C61B—C60B—H60B	120.0
C39—N3—C43	118.7 (4)	C59B—C60B—H60B	120.0
C39—N3—Pb2	118.8 (3)	C62B—C61B—C60B	120.0
C43—N3—Pb2	119.7 (3)	C62B—C61B—H61B	120.0
C44—N4—C48	119.4 (4)	C60B—C61B—H61B	120.0
C44—N4—Pb2	121.8 (3)	C61B—C62B—C57B	120.0
C48—N4—Pb2	118.3 (3)	C61B—C62B—H62B	120.0
O6—C25—O5	121.1 (4)	C57B—C62B—H62B	120.0

Symmetry codes: (i) −*x*, −*y*+1, −*z*+2; (ii) −*x*+1, −*y*+1, −*z*+1.

### Hydrogen-bond geometry (Å, °)

**Table:**

*D*—H···*A*	*D*—H	H···*A*	*D*···*A*	*D*—H···*A*
O10—H10···O5	0.85	1.83	2.670 (5)	171
O12A—H12C···O9^iii^	0.85	1.62	2.459 (9)	169
O12B—H12B···O4^iv^	0.85	1.81	2.612 (6)	158

Symmetry codes: (iii) *x*, *y*−1, *z*; (iv) −*x*, −*y*+1, −*z*+1.
